# Evaluation of Eosin-Methylene Blue as a Photosensitizer for Larval Control of *Aedes aegypti* by a Photodynamic Process

**DOI:** 10.3390/insects9030109

**Published:** 2018-08-30

**Authors:** Alessandra R. Lima, Cicera M. Silva, Cynthia S. A. Caires, Esmael D. Prado, Luciana R. P. Rocha, Isaias Cabrini, Eduardo J. Arruda, Samuel L. Oliveira, Anderson R. L. Caires

**Affiliations:** 1 Grupo de Óptica e Fotônica, Instituto de Física, Universidade Federal de Mato Grosso do Sul, CP 549, Campo Grande, MS 79070-900, Brazil; ramos.alessandra.09@gmail.com (A.R.L.); ciceraquimica@gmail.com (C.M.S.); samuel.oliveira@ufms.br (S.L.O.); 2 Faculdade de Ciências Exatas e Tecnologia, Universidade Federal da Grande Dourados, CP 533, Dourados, MS 79804-970, Brazil; EsmaelPrado@ufgd.edu.br (E.D.P.); LucianaPiovesan@ufgd.edu.br (L.R.P.R.); isaias_c04@yahoo.com.br (I.C.); ejarruda@gmail.com (E.J.A.); 3 Grupo de Espectroscopia e Bioinformática Aplicados a Biodiversidade e a Saúde, Faculdade de Medicina, CP 549, Campo Grande, MS 79070-900, Brazil; cynthiasuzyelen@yahoo.com.br

**Keywords:** *Aedes aegypti*, larval control, photodynamic process, eosin-methylene blue, sunlight

## Abstract

*Aedes aegypti* (*Ae. aegypti*) is a competent vector for transmitting important viral diseases such as yellow fever, dengue, chikungunya, and Zika. Several strategies have been applied to avoid *Ae. aegypti* proliferation by using environmental management, biological, and chemical approaches. However, the development of new methods for effective control of the insect vector population is still needed. Photodynamic control is an alternative way to control the vector population by using a physical approach based on the larval phototoxicity of a photosensitizer. In this context, the present study evaluated the use of eosin-methylene blue (EMB) as a new photosensitizer for photodynamic control of *Ae. aegypti* larval populations. The photodynamic assays were performed submitting *Ae. aegypti* third-instar larvae to different EMB concentrations (0.0, 0.5, 1.0, 5.0, 10.0, 50.0, and 100.0 µg mL^−1^) in combination of three different light doses (24.3, 48.6, and 97.2 J cm^−2^) under either white-light radiation from RGB LEDs or sunlight. The results demonstrated that EMB presented a rapid internalization into the larvae and was phototoxic. The photodynamic action induced 100% of larval mortality after about 40 min of sunlight irradiation even using low EMB concentration (0.5 µg mL^−1^). The findings reveal EMB as an effective photoactive compound to control larval populations of *Ae. aegypti* by photodynamic process induced by either sunlight or white-light from RGB LEDs.

## 1. Introduction

Viral diseases transmitted by mosquitoes are responsible for high rates of human mortality. Dengue fever, chikungunya, yellow fever, and Zika are among the most important viral diseases transmitted by *Aedes aegypti* (*Ae. aegypti*) [1,2,3]. Currently, chemical control is the most adopted strategy for limiting insect vector populations, especially by using organophosphate and pyrethroid insecticides [4]. However, new tools and strategies have been investigated, especially due to increasing pesticide resistance in *Ae. aegypti* [5,6,7,8]. Consequently, alternative insecticides obtained from plants, toxins from microorganisms, and new synthetic compounds have been researched, which ideally must present high efficiency, specificity, human safety, and lower environmental impact [9,10,11,12]. 

Therefore, in the present scenario, it is necessary to improve and develop alternative methods of controlling insecticide-resistant mosquitos. In this sense, photodynamic action is an emerging strategy for control of multidrug-resistant microorganism, killing the microorganism by producing singlet oxygen and/or reactive oxygen species (ROS) generated from the interaction between photoactive compounds (photosensitizers) and light in the presence of molecular oxygen. Although photodynamic control (PDC) of mosquitoes was first explored in 1928 [13], PDC has started to gain importance due to the limitations presented by the pesticide control methods, especially, because of growing concern about pesticide-resistant mosquitoes [14,15,16,17]. It is important to stress that, as a physical process, PDC has attracted attention because no microorganism resistance is expected to be developed during the photoinactivation treatment, and it can be applied to microorganism inactivation regardless of the multidrug resistance as well [18].

Barbieri reported larvicidal effect of xanthene derivatives in *Anopheles* and *Aedes* under white light irradiation; a mixture of Rose Bengal and Erythrosin was the most efficient for larval control, even in extremely dilute solutions [13]. Other studies have shown that porphyrin compounds such as photogem (hematoporphyrin derivative) and chlorophylls (chlorophyllins and pheophorbides) also exhibited larvicidal activity in *Ae. aegypti* when photoactivated [14,15,16,17]. To induce larval death by oxidative stress, a good photosensitizer (PS) should have an efficient singlet oxygen or strong ROS generation [19,20], be rapidly and selectively internalized or taken up by the larvae, be able to be activated by white light, and be rapidly eliminated from the environment [21].

In the present study, eosin-methylene blue according to Giemsa (EMB) was investigated as a photoactive compound for *Ae. aegypti* larval control by the photodynamic process, herein called as photodynamic control (PDC). Although EMB was developed more than 100 years ago by Gustav Giemsa (1867–1948) for staining of *Plasmodium*, the causative agent of malaria [22], its phototoxicity potential for microorganisms was only demonstrated recently [23,24,25,26]. The studies revealed that EMB under red-light irradiation was able to photoinactivate Gram-positive (ATCC 25923) and Gram-negative (ATCC 25922) bacteria as well as multidrug-resistant *mcr-1* positive *Escherichia coli* (CCBH 23595) [26].

## 2. Materials and Methods

### 2.1. Maintenance of Larvae

*Ae. aegypti* eggs (Rockefeller strain from Laboratory of Insect Vectors of Federal University of Grande Dourados) were maintained in plastic trays with tap water at 25 °C. After egg hatching, the larvae were fed daily with 1 mg/larva of fish ration (Alcon^®^, Camboriú, Santa Catarina, Brazil) until reaching the third-instar larval stage (L_3_) [27]. The larvae were picked up with a disposable Pasteur pipette to remove the excess of water. Then, they were put on a Petri dish (20 larvae per dish) with a depth of 15 mm, containing 20 mL of PS solution.

### 2.2. Photosensitizer

Eosin-methylene blue according to Giemsa (EMB) was the PS evaluated. EMB is a dye commonly used for microscopy analysis and prepared from a combination of Azure II (a mixture of Azure I with an equal amount of methylene blue) and eosinate of Azure II (an equimolar combination of Azure I, methylene blue, and Y-eosin) [28,29]. The EMB solution was prepared by diluting EMB powder (99% of purity, Vetec^®^, Rio de Janeiro, Brazil) with distilled water, obtaining seven concentrations (0.0, 0.5, 1.0, 5.0, 10.0, 50.0, and 100.0 µg mL^−1^).

### 2.3. Optical Characterization

The light absorption profile of EMB in the 250–800 nm range was determined at room temperature using a Cary 50 UV-Vis (Varian^®^, Palo Alto, CA, USA) spectrophotometer. EMB was diluted in distilled water at 35.0 µg mL^−1^. The measurement was performed using a quartz cell with 10 mm optical path.

### 2.4. Internalization Time

The time of internalization (TI) of the PS into the larvae was determined by optical microscopy analysis. Initially, 30 larvae were placed on a Petri dish containing a PS solution at 10.0 µg mL^−1^; then, for 45 min, three larvae were collected every 5 min for the optical image analysis. The larval images were recorded, immediately after collecting the larvae from the Petri dish, using a trinocular microscope (Opton^®^, São Paulo, Brazil), a TA-0124 digital camera, and TSView 7.1.1.7 software.

### 2.5. Bioassays with Ae. aegypti

**Figure 1:**
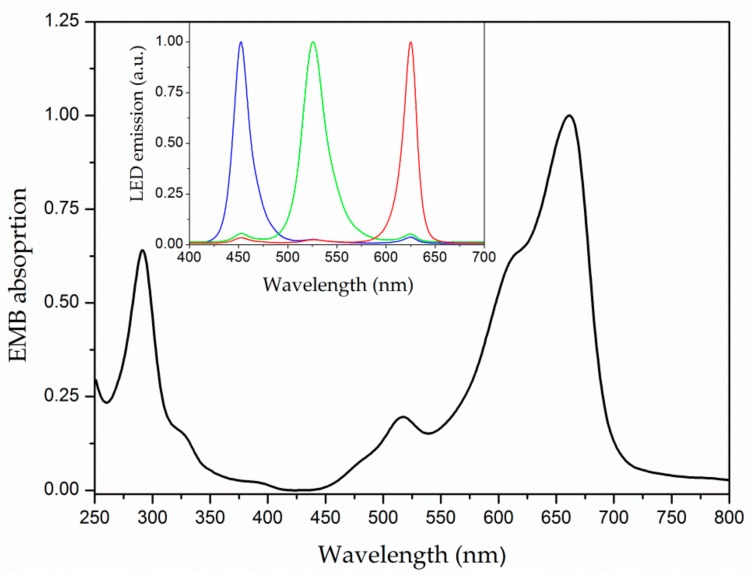
Normalized absorption spectrum of eosin-methylene blue (EMB) diluted in distilled water. Inset: RGB LED emission spectra.

The PDC assays were carried out with *Ae. aegypti* third-instar larvae in Petri dishes. The larvae remained immersed in PS aqueous solution for 120 min before PDC experiments. For each tested PS concentration, the larvae were separated in two main groups: irradiated and non-irradiated (dark) groups. The irradiation was performed using white light provided by a customized RGB LED device, which allows the appropriate combination of red (625 nm), green (525 nm), and blue (450 nm) radiation, as presented in the inset of Figure 1. Firstly, three different illumination conditions were tested for the irradiated groups, maintaining the illuminance at 27 mW/cm^2^ (originated from 9 mW/cm^2^ at 450 nm, 535 nm, and 625 nm) and varying the irradiation time (15, 30, and 60 min). Larval mortality was assessed as a function of time after finishing light irradiation. The efficacy of the PDC of larvae was also investigated using sunlight irradiation (~135,000 lx, which is equivalent to 20 mW/cm^2^ at 555 nm). In this assay, the Petri dishes containing the larvae of the control group (non-irradiated) were kept covered by a black cover in the natural environment, avoiding sunlight exposure. In the sunlight-based experiments, the mortality of the larvae was monitored during the sun exposure. For both experiments, a larva was considered dead when it was not moving after stimulus with a Pasteur pipette [30]. Finally, a positive control was performed by using a conventional larvicide (Temephos) to determine whether EMB is chemically toxic to the larvae. All tests were done in triplicate for each experimental group, from which the mean value, standard deviation (SD), and standard error (SE) were calculated.

## 3. Results and Discussion

### 3.1. Optical Characterization

To characterize how EMB can be light-activated, the optical absorption of EMB was investigated. EMB presents a wide absorption spectrum in the ultraviolet and visible range. Figure 1 reveals that EMB presents two main absorption peaks in the 250–350 and 580–700 nm ranges. In addition, a well-defined absorption band in the blue-green range (450–550 nm) is also observed. Therefore, EMB can absorb UV and visible light provided by either sunlight or white-light from RGB LEDs.

### 3.2. Internalization Time

**Figure 2:**
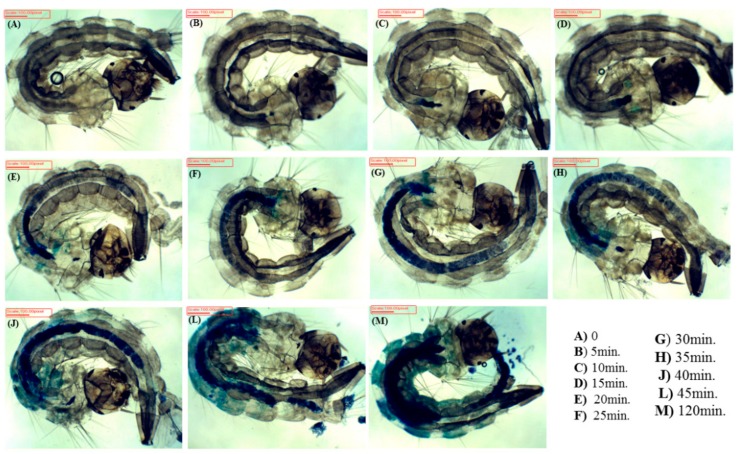
Images of *Aedes aegypti* larvae after their immersion in photosensitizer (PS) aqueous solution of EMB as a function of immersion time.

EMB was rapidly internalized into larvae (Figure 2). After a few minutes of immersion of the larvae in the PS solution, EMB was already in the digestive system. The PS completely filled their digestive system after about 30 min, translocating radially to other parts of the larvae. The larvae began to excrete PS after 45 min, reaching a steady state of PS concentration after 120 min, when PS was spread all over the larvae.

### 3.3. Photodynamic Control of Ae. aegypti Larvae

#### 3.3.1. White-Light Radiation from RGB LEDs

**Figure 3:**
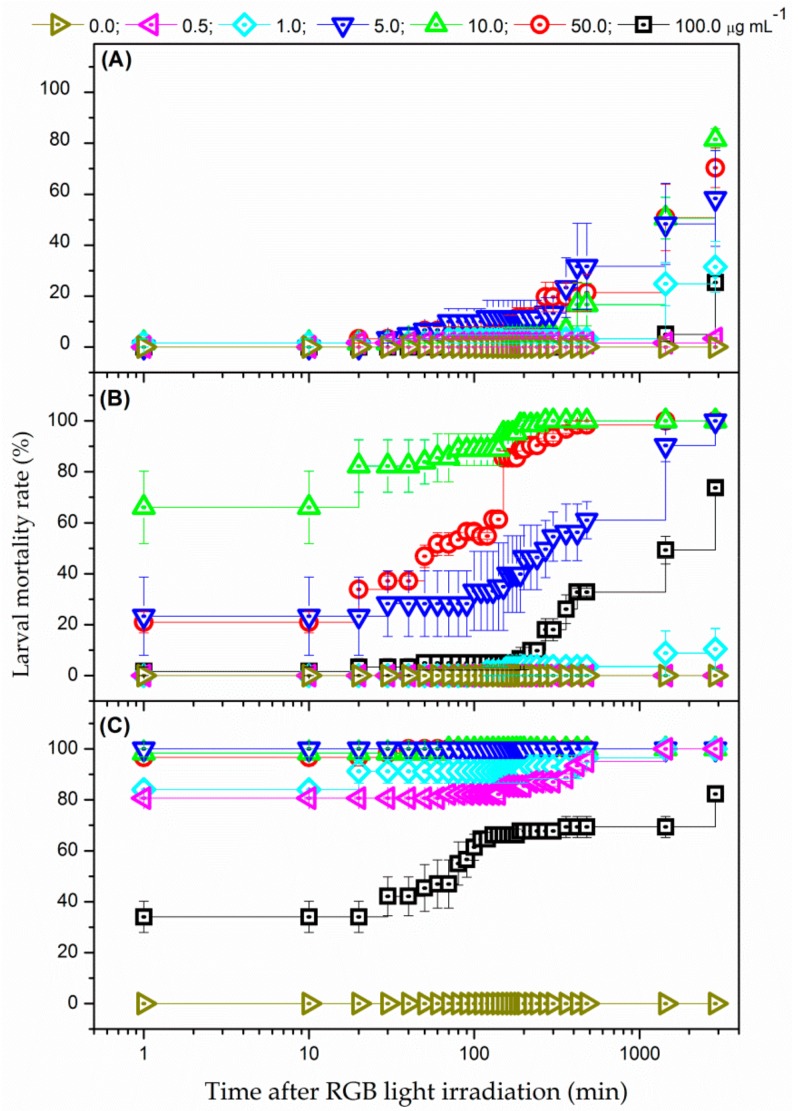
Larval mortality rate as a function of time after white-light irradiation from RGB LEDs at 27 mW/cm^2^ during: (**A**) 15, (**B**) 30, and (**C**) 60 min, for different EMB concentrations: (δ) 0.0, (Ψ) 0.5, (N) 1.0, (Δ) 5.0, (9) 10.0, (.) 50.0, and (#) 100.0 µg mL^−1^. Error bars represent SE.

Figure 3 shows the mortality rates for larvae of *Ae. aegypti* after irradiation using white-light provided by RGB LEDs and submitted to EMB concentration of 0.0 (no PS—negative control), 0.5, 1.0, 5.0, 10.0, 50.0, and 100.0 µg mL^−1^. Three different illumination conditions (24.3, 48.6, 97.2 J cm^−2^) were employed. The larval mortality rate was illumination and PS concentration dependent. For instance, while no mortality was observed for all tested PS concentrations up to 20 min after concluded irradiation of 24.3 J cm^−2^, a maximum mortality rate of 81.5% was obtained after 2880 min (48 h) using the same illumination time with immersion in a PS solution with 10.0 µg mL^−1^. Likewise, the mortality rate of 100% of larvae subjected to PS concentration of 5.0 µg mL^−1^ was verified just after the conclusion of the irradiation of 97.2 J cm^−2^ (i.e., the larvae death occurred during the irradiation process).

Additionally, the data also revealed that the optimal concentration range of EMB remained between 5.0–50.0 µg m^−1^ for PDC of *Ae. aegypti* larvae using white-light from RGB LEDs, depending on the illumination time. This finding could be related to the low number of PS molecules internalized into the larvae for the PS concentrations lower than 5.0 µg mL^−1^ as well as the reduction of light intensity through the solution for PS concentrations over 50.0 µg mL^−1^ because of the inner filter effect, hindering the PS photoactivation in deeper region of the medium [31].

The findings also revealed that the illumination process was not toxic for the larvae themselves as no larval death was observed in the negative control samples (no PS) for the tested light setups. In addition, EMB did not induce larval death for all tested PS concentrations when the larvae were kept in the dark (non-irradiated group). Here, it is worth noting that Temephos (positive control) induced 100% of larval mortality at a very low concentration (0.12 µg mL^−1^) as presented in Figure S1 in the Supplementary Materials. These results reveal that EMB was non-toxic to the larvae even when used at a higher concentration (100.0 µg mL^−1^) than Temephos. Although Ahmed et al. reported that methylene blue and eosin may be chemically toxic to *Spodoptera littoralis* larvae, larval death was only observed when using high concentrations of the chemical compound [32]. They determined that eosin and methylene blue induced the death of 50% of the larval population at a concentration of 884 and 483 µg mL^−1^, respectively [32].

#### 3.3.2. Sunlight Irradiation

**Figure 4:**
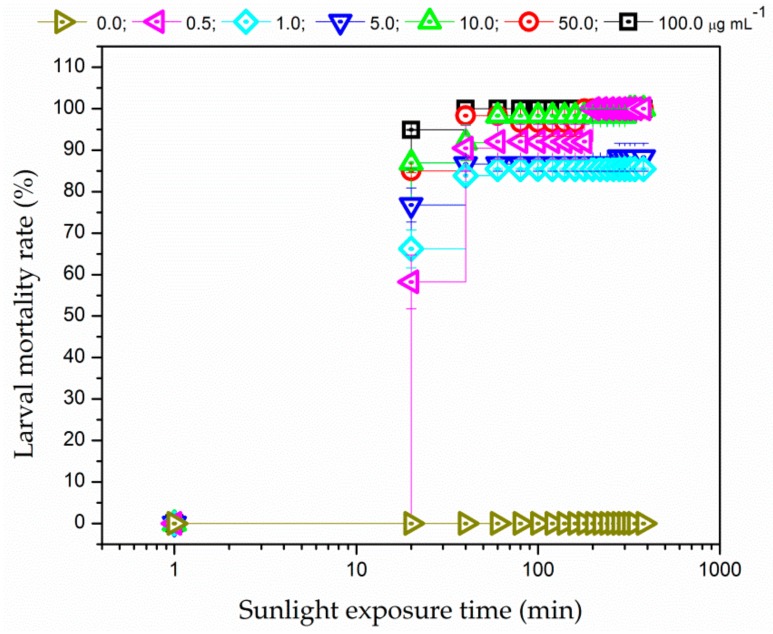
Larval mortality rate as a function of exposure time to solar irradiation of 135,000 lx for different EMB concentrations: (δ) 0.0, (Ψ) 0.5, (N) 1.0, (Δ) 5.0, (9) 10.0, (.) 50.0, and (#) 100.0 µg mL^−1^. Error bars represent SE.

PDC of the larvae was also investigated using sunlight as the radiation source. Figure 4 shows that the larval mortality rate was higher than 50% during 20 min of sunlight exposure, regardless of PS concentrations. EMB induced rapid larval death even at low concentration (0.5 µg mL^−1^). Furthermore, unlike the results obtained when using RGB LEDs as light source, 100% larval mortality was promoted for the highest PS concentration (100.0 µg mL^−1^). Therefore, EMB is a good candidate to be photoactivated in larval PDC applications not only with white-light from RGB LEDs but also with sunlight.

The higher mortality rates obtained under sun illumination could be explained by the fact that a higher light illuminance was achieved with solar radiation when compared with one provided by the RGB LEDs. Moreover, sunlight radiation provides greater spectral overlap with respect to the EMB absorption spectrum than one achieved using RGB LEDs so that additional channels of ROS production could not be ruled out. In addition, no mortality was observed in the larvae immersed in EMB solution and kept in the dark at the same environmental condition (non-irradiated control groups) as well as in the larvae exposed to solar radiation in the absence of the EMB (irradiated control group). 

As a proof of principle, these results demonstrated that EMB can be used for controlling the larval population. Although the experiments were carried out in a non-natural breeding habitat (Petri dishes), our results indicate that PDC may potentially be applied in deep-water breeding sites because the larvae need to reach the water (breeding) surface to breath. In addition, results also revealed in the laboratory experiments that PDC induced larval death when a low light dose was used (i.e., irradiating for 15 min at 27 mW/cm^2^), suggesting that PDC can induce larval death even in shaded breeding sites. Even though the present findings indicate that EMB may be successfully applied for larval PDC, further investigations are still needed to test the effectiveness of larval PDC in the natural environment and conditions as well as to determine whether the EMB can be safely used in the environment. 

## 4. Conclusions

The photodynamic control of *Ae. aegypti* larvae immersed in EMB solution and subsequently light-irradiated was assessed. Results revealed that EMB is phototoxic to *Ae. aegypti* larvae even at low concentration (0.5 µg mL^−1^). EMB presented a fast internalization time into the larvae, and it was able to induce a rapid and efficient larval mortality using either sunlight or irradiation from a white-light source. The results revealed that 100% larval mortality was achieved when the larvae were exposed to sunlight (about 40 min), while EMB was non-toxic to the larvae in the dark. In summary, this study demonstrated as a proof of principle that EMB has great potential to be applied in PDC of the larval population of *Ae. aegypti* under sunlight exposure.

## Supplementary Materials

The following are available online at http://www.mdpi.com/2075-4450/9/3/109/s1, Figure S1: Larval mortality rate as a function of time induced by Temephos at different concentrations: (#) 0.012, (.) 0.12, (9) 0.5, (Δ) 1.0 µg mL^−1^. Error bars represent SE.

## References

[1] World Health Organization, Regional Office for South-East Asia (2009). Dengue: Guidelines for Diagnosis, Treatment, Prevention, and Control.

[2] World Health Organization (2015). Zika virus outbreaks in the Americas. Wkly. Epidemiol. Rec..

[3] World Health Organization (2012). Global Strategy for Dengue Prevention and Control. 2012–2020.

[4] Ministério da Saúde (2009). Diretrizes Nacionais Para a Prevenção e Controle de Epidemias de Dengue.

[5] Bellinato D.F., Viana-Medeiros P.F., Araújo S.C., Martins A.J., Lima J.B.P., Valle D. (2016). Resistance Status to the Insecticides Temephos, Deltamethrin, and Diflubenzuron in Brazilian *Aedes aegypti* Populations. BioMed Res. Int..

[6] Smith L.B., Kasai S., Scott J.G. (2016). Pyrethroid resistance in *Aedes aegypti* and *Aedes albopictus*: Important mosquito vectors of human diseases. Pestic. Biochem. Physiol..

[7] Haddi K., Tomé H.V.V., Du Y., Valbon W.R., Nomura Y., Martins G.F., Dong K., Oliveira E.E. (2017). Detection of a new pyrethroid resistance mutation (V410L) in the sodium channel of *Aedes aegypti*: A potential challenge for mosquito control. Sci. Rep..

[8] Moyes C.L., Vontas J., Martins A.J., Ng L.C., Koou S.Y., Dusfour I., Raghavendra K., Pinto J., Corbel V., David J.P. (2017). Contemporary status of insecticide resistance in the major *Aedes* vectors of arboviruses infecting humans. PLoS Negl. Trop. Dis..

[9] Grzybowski A., Tiboni M., Silva M.A., Chitolina R.F., Passos M., Fontana J.D. (2013). Synergistic larvicidal effect and morphological alterations induced by ethanolic extracts of *Annona muricata* and *Piper nigrum* against the dengue fever vector *Aedes aegypti*. Pest. Manag. Sci..

[10] Arruda E.J., Rossi A.P.L., Porto K.R.A., Oliveira L.C.S., Arakaki A.H., Scheidt G.N., Soccol C.R. (2010). Evaluation of toxic effects with transition metal ions, EDTA, SBTI and acrylic polymers on *Aedes aegypti* (L., 1762) (Diptera: Culicidae) and *Artemia salina* (Artemidae). Braz. Arch. Biol. Technol..

[11] Boethling R.S., Sommer E., DiFiore D. (2007). Designing Small Molecules for Biodegradability. Chem. Rev..

[12] Coelho J.S., Santos N.D.L., Napoleão T.H., Gomes F.S., Ferreira R.S., Zingali R.B., Coelho L.C.B.B., Leite S.P., Navarro D.M.A.F., Paiva P.M.G. (2009). Effect of *Moringa oleifera* lectin on development and mortality of *Aedes aegypti* larvae. Chemosphere.

[13] Barbieri A. (1928). Sensibilizadores fluorescentes como larvicidas. Accion fotodinamica de la luz. Riv. Malariol..

[14] Amor T.B., Jori G. (2000). Sunlight-activated insecticides: Historical background and mechanisms of phototoxic activity. Insect Biochem. Mol. Biol..

[15] Azizullah A., Rehman Z.U., Ali I., Murad W., Muhammad N., Ullah W., Häder D.P. (2014). Chlorophyll derivatives can be an efficient weapon in the fight against dengue. Parasitol. Res..

[16] Souza L.M., Inada N.M., Pratavieira S., Corbi J.J., Kurachi C., Bagnato V.S. (2017). Efficacy of Photogem^®^ (Hematoporphyrin Derivative) as a Photoactivatable Larvicide against *Aedes aegypti* (Diptera: Culicidae) Larvae. J. Life Sci..

[17] Lucantoni L., Magaraggia M., Lupidi G., Ouedraogo R.K., Coppellotti O., Esposito F., Fabris C., Jori G., Habluetzel A. (2011). Novel, Meso-Substituted Cationic Porphyrin Molecule for Photo-Mediated Larval Control of the Dengue Vector *Aedes aegypti*. PLoS Negl. Trop. Dis..

[18] Grinholc M., Rapacka-Zdonczyk A., Rybak B., Szabados F., Bielawski K.P. (2014). Multiresistant Strains Are as Susceptible to Photodynamic Inactivation as Their Naı¨ve Counterparts: Protoporphyrin IX-Mediated Photoinactivation Reveals Differences between Methicillin-Resistant and Methicillin-Sensitive *Staphylococcus aureus* Strains. Photomed. Laser Surg..

[19] Song R., Feng Y., Wang D., Xu Z., Li Z., Shao Z. (2017). Phytoalexin Phenalenone Derivatives Inactivate Mosquito Larvae and Root-knot Nematode as Type-II Photosensitizer. Sci. Rep..

[20] Hamblin M.R. (2016). Antimicrobial photodynamic inactivation: A bright new technique to kill resistant microbes. Curr. Opin. Microbiol..

[21] Dondji B., Duchon S., Diabate A., Herve J.P., Corbel V., Hougard J.-M., Santus R., Schrevel J. (2005). Assessment of Laboratory and Field Assays of Sunlight-Induced Killing of Mosquito Larvae by Photosensitizers. J. Med. Entomol..

[22] Fleischer B. (2004). Editorial: 100 years ago: Giemsa’s solution for staining of *plasmodia*. Trop. Med. Int. Health.

[23] Lima A.R., Silva C.M., Caires C.S.A., Nascimento V.A., Rocha L.R.P., Cabrini I., Arruda E.J., Oliveira S.L., Caires A.R.L. (2017). Photodynamic control of *Aedes aegypti* larvae (Diptera: Culicidae). Photodiagn. Photodyn. Ther..

[24] Caires C.S.A., Leal C.R.B., Ramos C.A.N., Lima A.R., Caires A.R.L., Arruda E.J., Oliveira S.L., Nascimento V.A. (2017). Photodynamic antimicrobial therapy on *S*. *aureus* and *E*. *coli* by using Giemsa stain as photosensitizer. Photodiagn. Photodyn. Ther..

[25] Caires C.S.A., Leal C.R.B., Ramos C.A.N., Bogo D., Lima A.R., Arruda E.J., Oliveira S.L., Caires A.R.L., Nascimento V.A. (2017). Photoinactivation effect of eosin methylene blue and chlorophyllin sodium-copper against *Staphylococcus aureus* and *Escherichia coli*. Lasers Med. Sci..

[26] Caires C.S.A., Leal C.R.B., Rodrigues A.C.S., Ramos C.A.N., Chang M.R., Lima A.R., Arruda E.J., Oliveira S.L., Nascimento V.A., Caires A.R.L. (2018). Photoinactivation of mcr-1 positive *Escherichia coli*. Laser Phys. Lett..

[27] Arrivillaga J., Barrera R. (2004). Food as a limiting factor for *Aedes aegypti* in water-storage containers. J. Vector Ecol..

[28] Barcia J.J. (2007). The Giemsa Stain: Its History and Applications. Int. J. Surg. Pathol..

[29] Brammer S.P., Toniazzo C., Poersch L.B. (2015). Corantes comumente empregados na citogenética vegetal. Arq. Inst. Biol. (Sao Paulo).

[30] Campos J., Andrade C.F.S. (2002). Insecticide resistance in *Simulium* populations (Diptera, Simuliidae). Cadernos Saude Publica.

[31] Lakowicz J.R. (2006). Principles of Fluorescence Spectroscopy.

[32] Ahmed Y.M., Mostafa A.M.A., Elewa M.A. (1985). Toxicity of certain dyes as insecticides and their joint action with some pyrethroids. J. Environ. Sci. Health Part B.

