# Supplementary material for: Evaluation of Eosin-Methylene Blue as a Photosensitizer for Larval Control of Aedes aegypti by a Photodynamic Process
Source: Insects. 2018 Aug 30;9(3):109. doi: 10.3390/insects9030109 (PMC6163889; doi:10.3390/insects9030109)
Supplement: Supplementary file 1 [file insects-09-00109-s001.pdf]

Supporting Information

Evaluation of Eosin-Methylene Blue as a Photosensitizer for Larval Control of *Aedes aegypti* by a Photodynamic Process

Alessandra R. Lima [1,2], Cicera M. Silva [1,2], Cynthia S. A. Caires [3], Esmael D. Prado [2], Luciana R. P. Rocha [2], Isaias Cabrini [2], Eduardo J. Arruda [2], Samuel L. Oliveira [1] and Anderson R. L. Caires [1,*]

[1] Grupo de Óptica e Fotônica, Instituto de Física, Universidade Federal de Mato Grosso do Sul, CP 549, 79070-900 Campo Grande, MS, Brazil
[2] Faculdade de Ciências Exatas e Tecnologia, Universidade Federal da Grande Dourados, CP 533, 79804-970 Dourados, MS, Brazil
[3] Grupo de Espectroscopia e Bioinformática Aplicados a Biodiversidade e a Saúde, Faculdade de Medicina, CP 549, 79070-900 Campo Grande, MS, Brazil
* Correspondence: anderson.caires@ufms.br; Tel.: +55-67-3345-7030

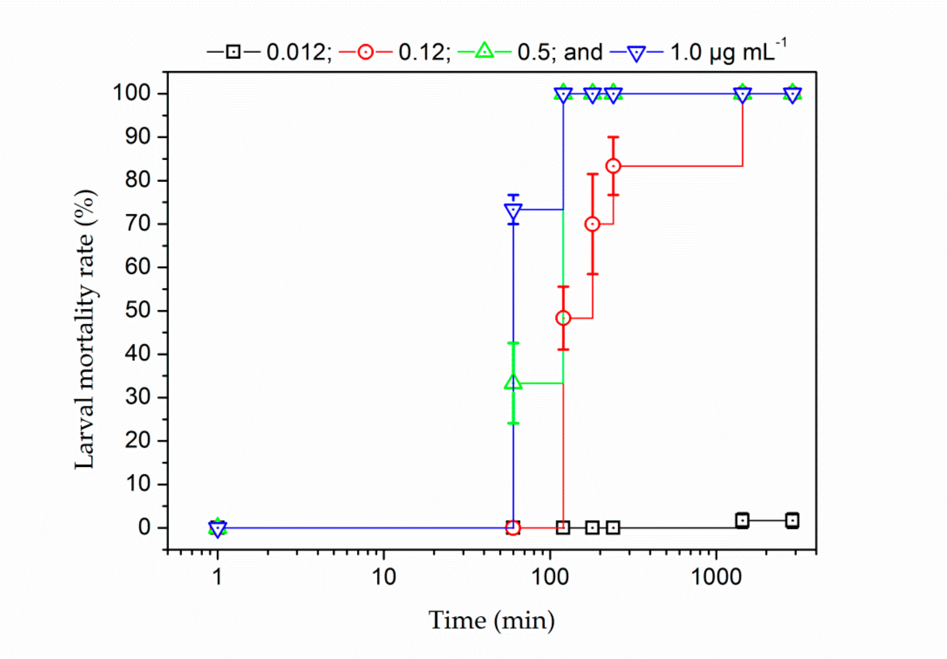

Figure S1. Larval mortality rate as a function of time induced by Temephos at different concentrations: (#) 0.012, (.) 0.12, (9) 0.5, (Δ) 1.0 μg mL^{-1}. Error bars represent SE.
